# Supplementary material for: An acetate electrolyte for enhanced pseudocapacitve capacity in aqueous ammonium ion batteries
Source: Nat Commun. 2024 Mar 2;15:1934. doi: 10.1038/s41467-024-46317-5 (PMC10908845; doi:10.1038/s41467-024-46317-5)
Supplement: Supplementary file 1 — Supplementary information [file 41467_2024_46317_MOESM1_ESM.pdf]

Supplementary Information

An acetate electrolyte for enhanced pseudocapacitve capacity in aqueous ammonium ion batteries

Zhuoheng Bao[1], Chengjie Lu[1], Qiang Liu[1], Fei Ye[1], Weihuan Li[1], Yang Zhou[1], Long Pan[1], Lunbo Duan[2], Hongjian Tang[2], Yuping Wu[2], Linfeng Hu[1]*, ZhengMing Sun[1]*

[1] School of Materials Science and Engineering, Southeast University, Nanjing, 211189, China

These authors contributed equally: Zhuoheng Bao, Chengjie Lu

Address correspondence to: linfenghu@seu.edu.cn; zmsun@seu.edu.cn

[2] School of Energy and Environment, Southeast University, Nanjing 211189, P. R. China

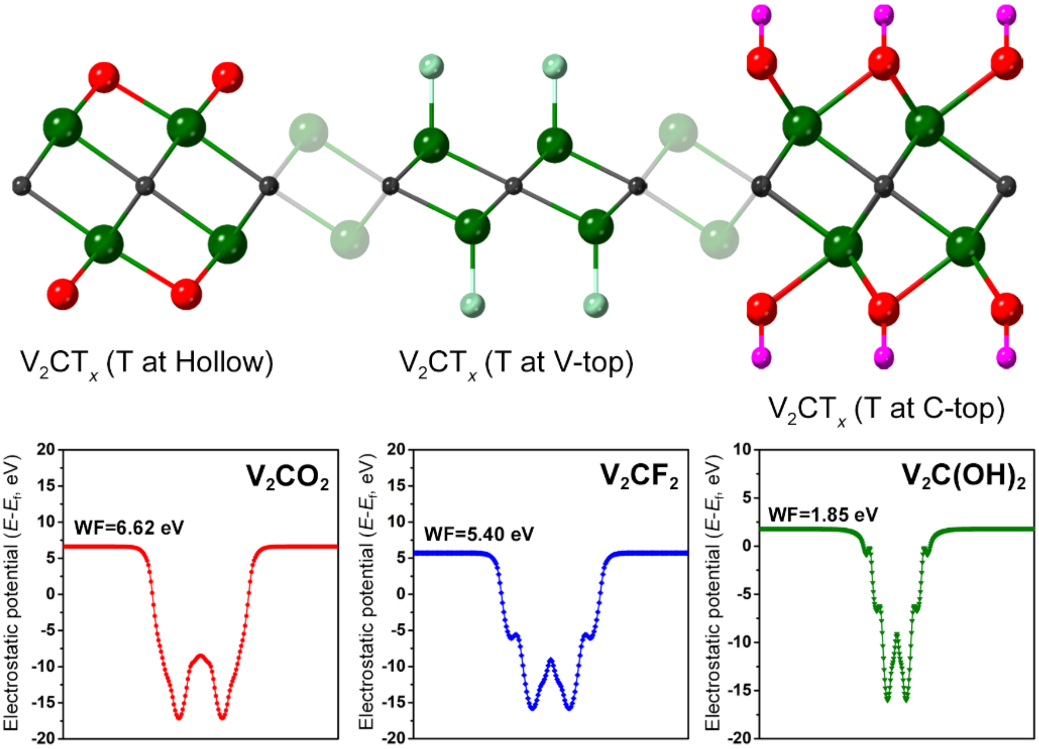

Supplementary Fig. 1 Possible positions of surface terminations on V_2CT_x: Hollow, V-top and C-top together with the computation results of electrostatic potential of V_2CT_x (T=-O, -F, -OH) at Hollow site (energetically preferred, ground state).

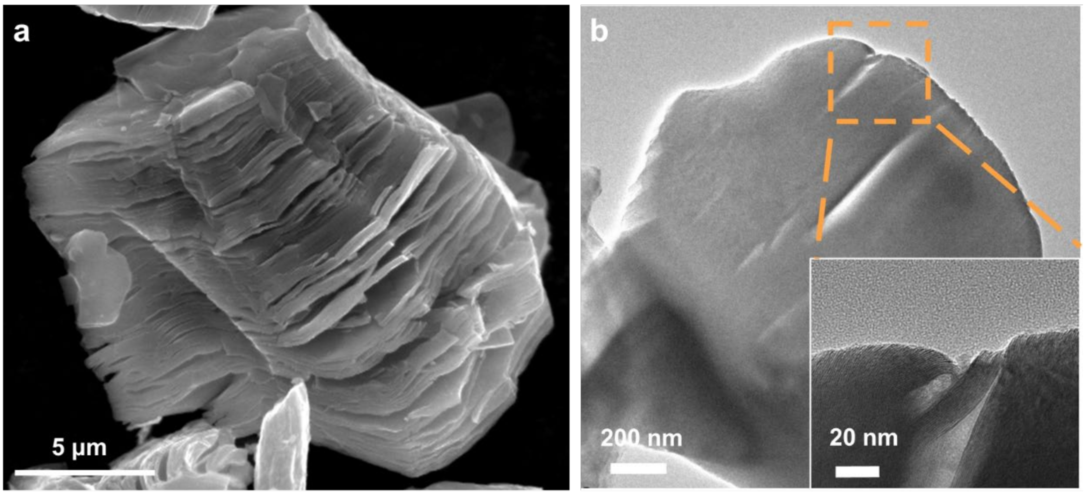

Supplementary Fig. 2 a) SEM and b) TEM images of m-V_2CT_x.

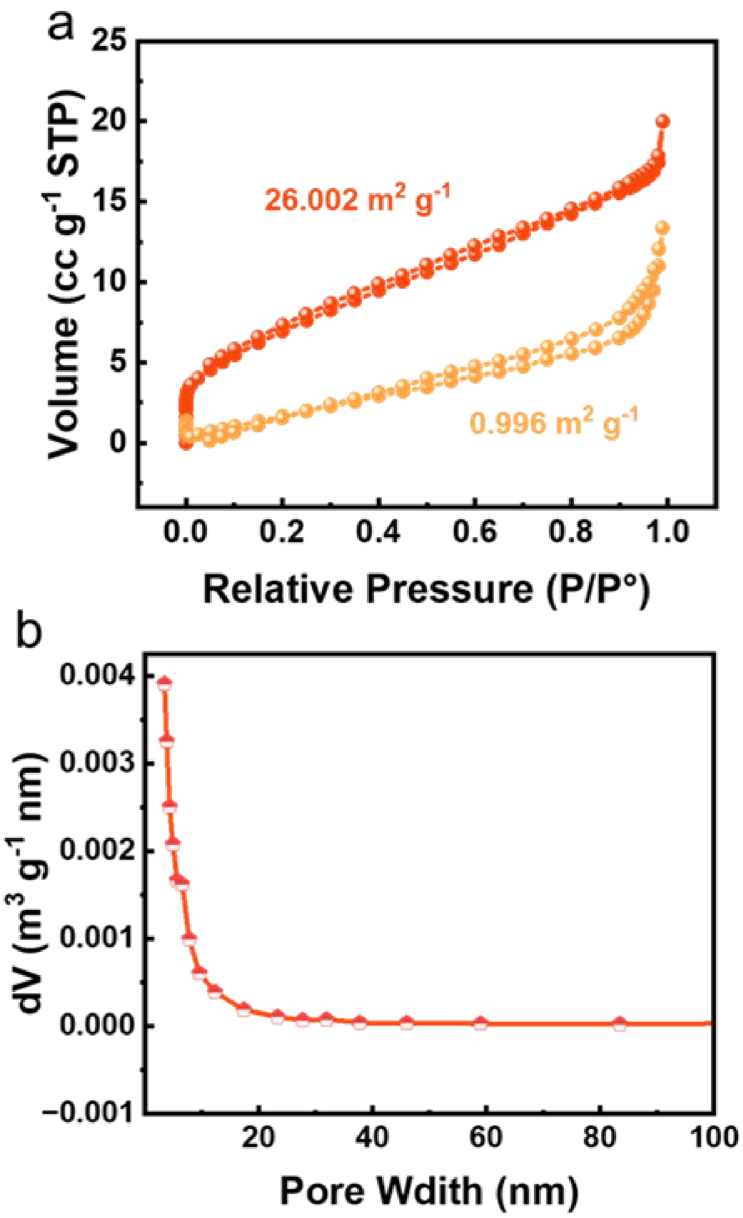

Supplementary Fig. 3 a) N_2 adsorption/desorption isotherms of m-V_2CT_x (orange line) and d-V_2CT_x (red line). b) Pore size distribution curve of d-V_2CT_x.

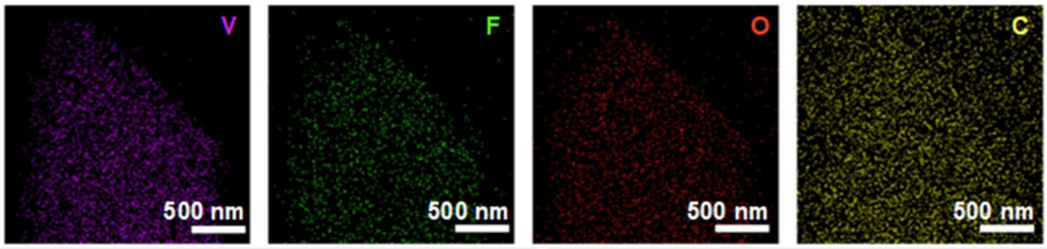

Supplementary Fig. 4 Elemental mappings of V (purple), F (green), O (red), C (yellow) captured on d-V_2CT_x nanosheet, respectively.

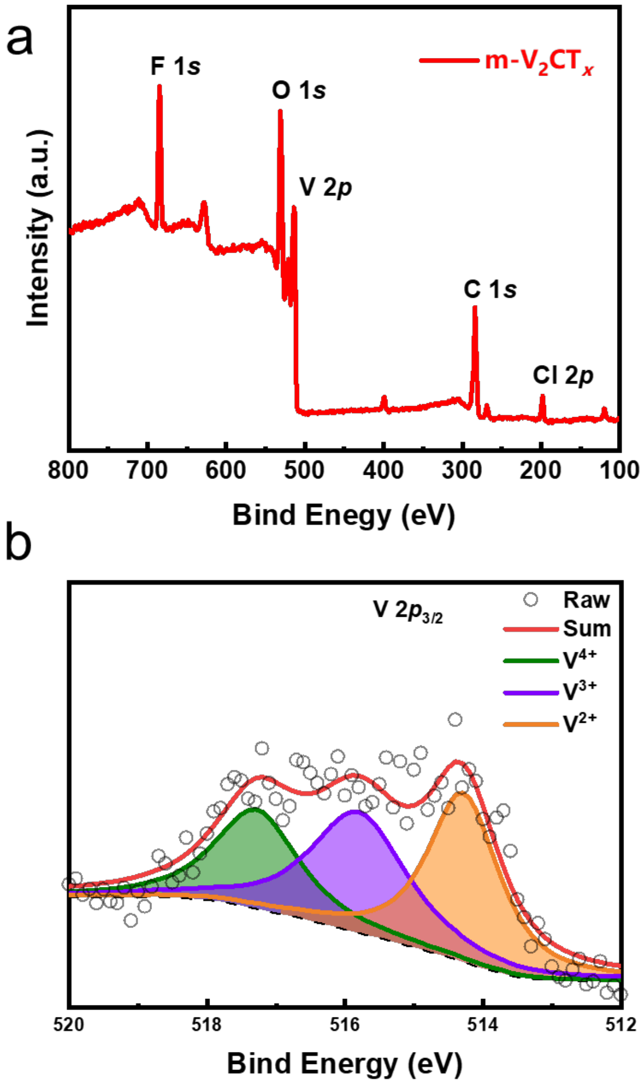

Supplementary Fig. 5 a) XPS characterization and b) the high-resolution V $2p_{3/2}$ XPS spectra of d-V_2CT_x.

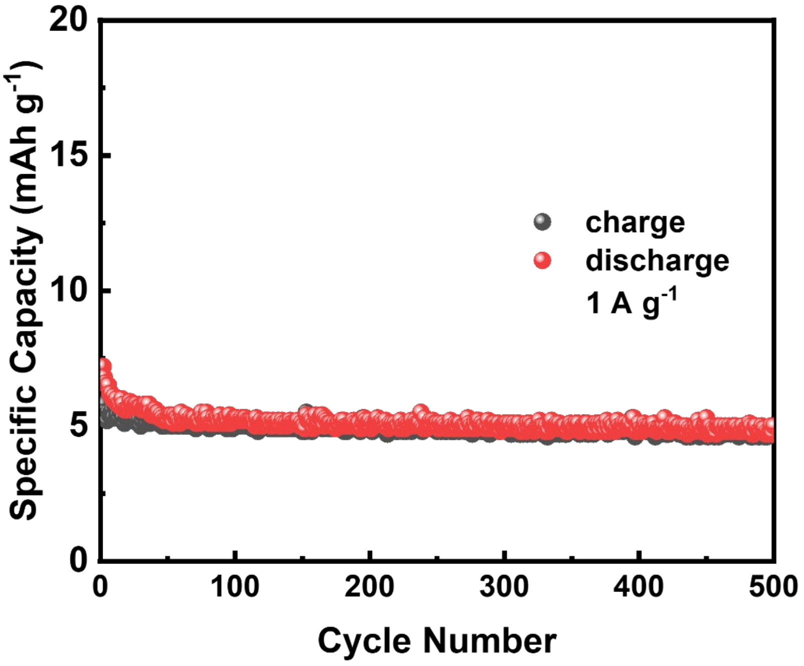

Supplementary Fig. 6 Specific capacity of V_2AlC sample in 0.5 M NH_4Ac at 1 A g^{-1}.

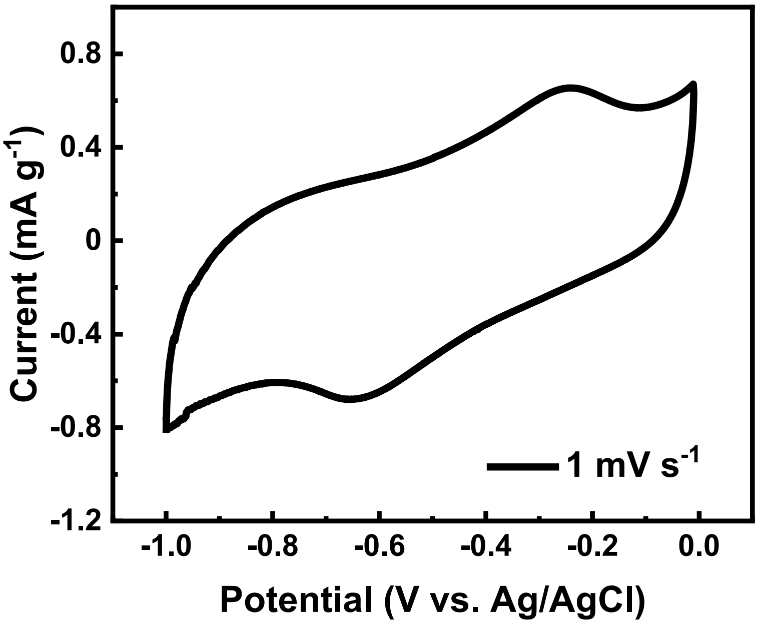

Supplementary Fig. 7 Cyclic voltammetry curve of d-V_2CT_x in 0.5 M NH_4Ac electrode at 1 mV s^{-1}.

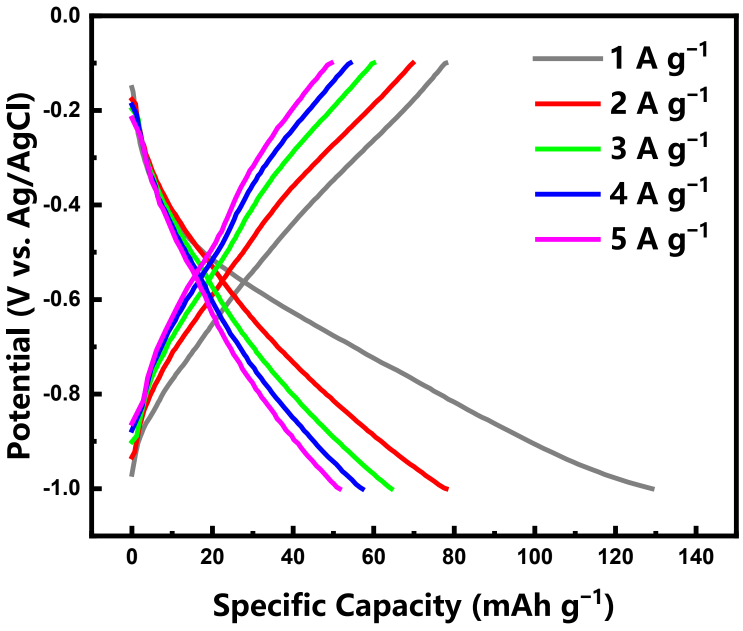

Supplementary Fig. 8 the galvanostatic discharge/charge profiles of d-V_2CT_x in 0.5 M NH_4Ac at different current densities.

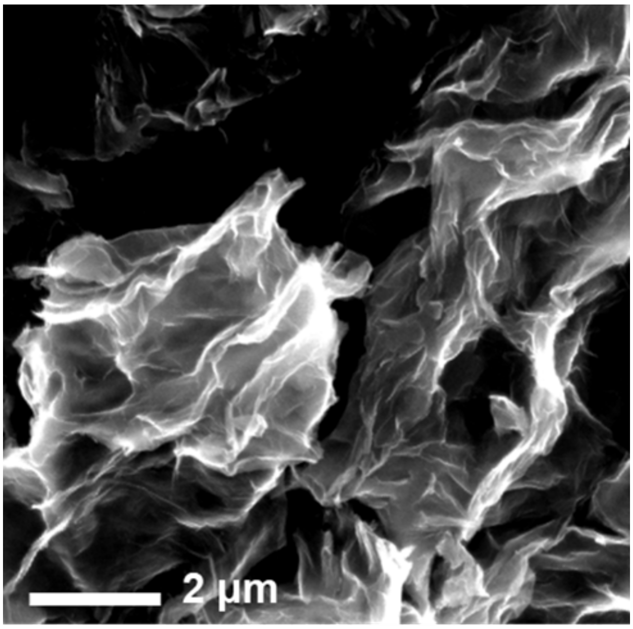

Supplementary Fig. 9 SEM morphology of d-V_2CT_x after 500 cycles in 0.5 M NH_4Ac at 1 A g^{-1}.

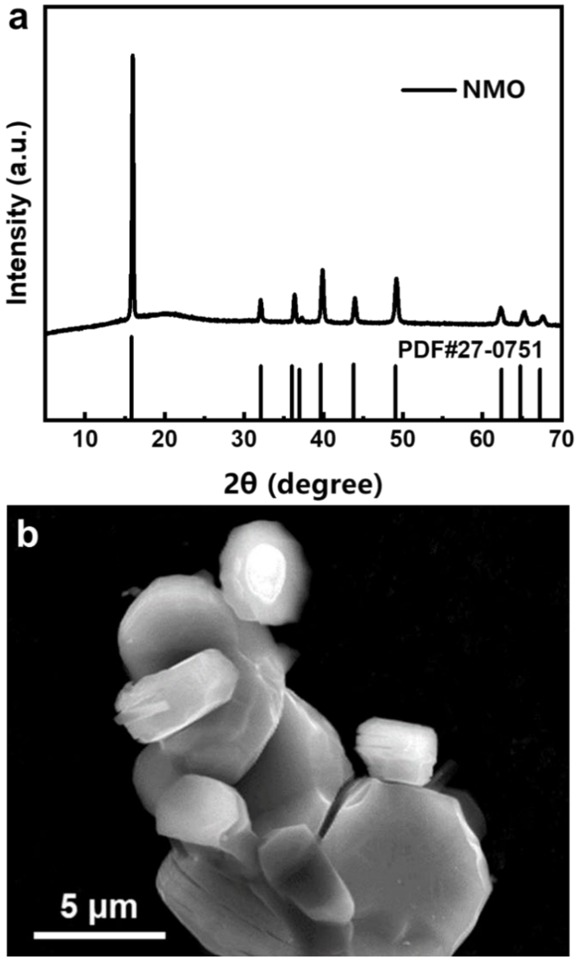

Supplementary Fig. 10 a) XRD pattern of $Na_{0.6}MnO_2$ (NMO). b) SEM image of NMO particles with a size of ~5 μm.

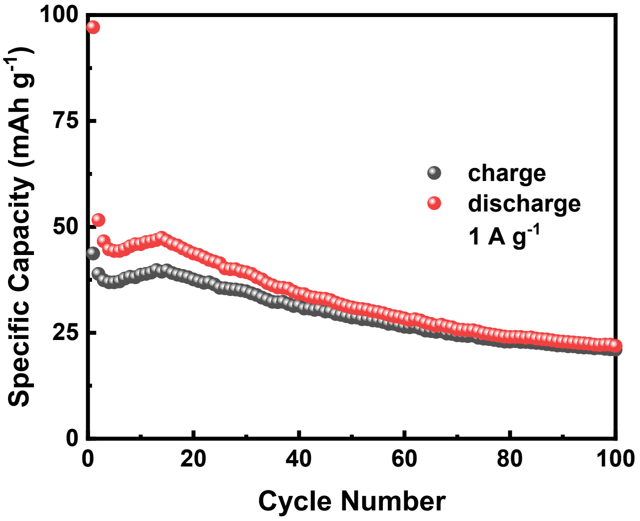

Supplementary Fig. 11 Cycling performance of NMO material in 0.5 M NH_4Ac at 1 A g^{-1}.

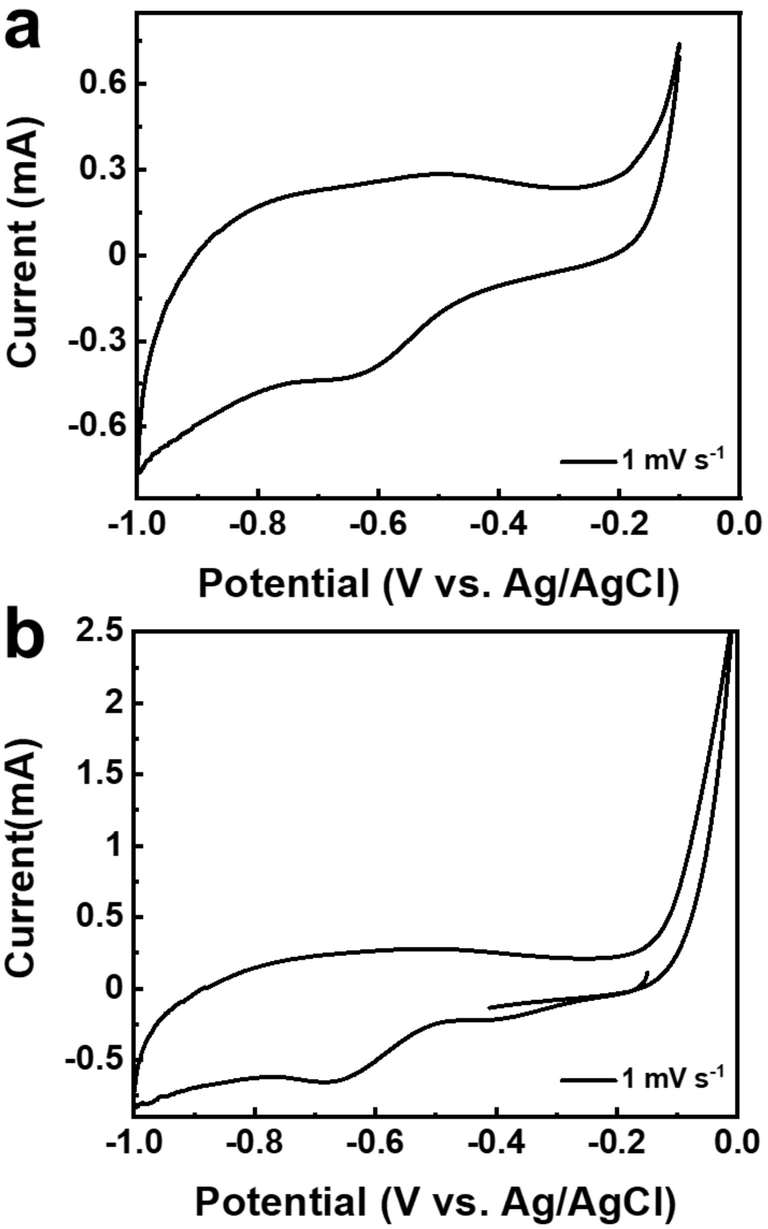

Supplementary Fig. 12 CV curves of d-V_2CT_x in (a) 10M and (b) 20M NH_4Ac electrolyte.

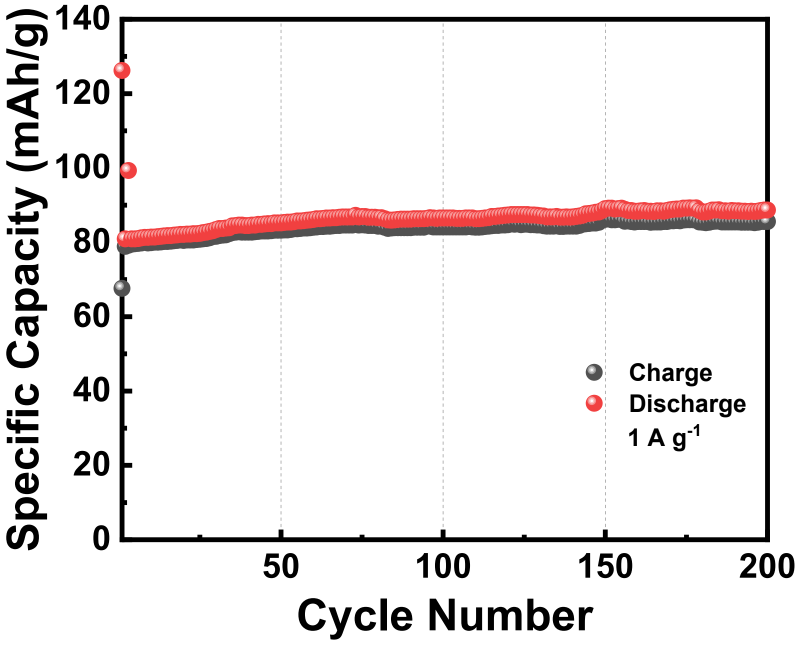

Supplementary Fig. 13 Cycling performance of d-V_2CT_x electrode in 20 M NH4Ac at 1 A g^{-1}.

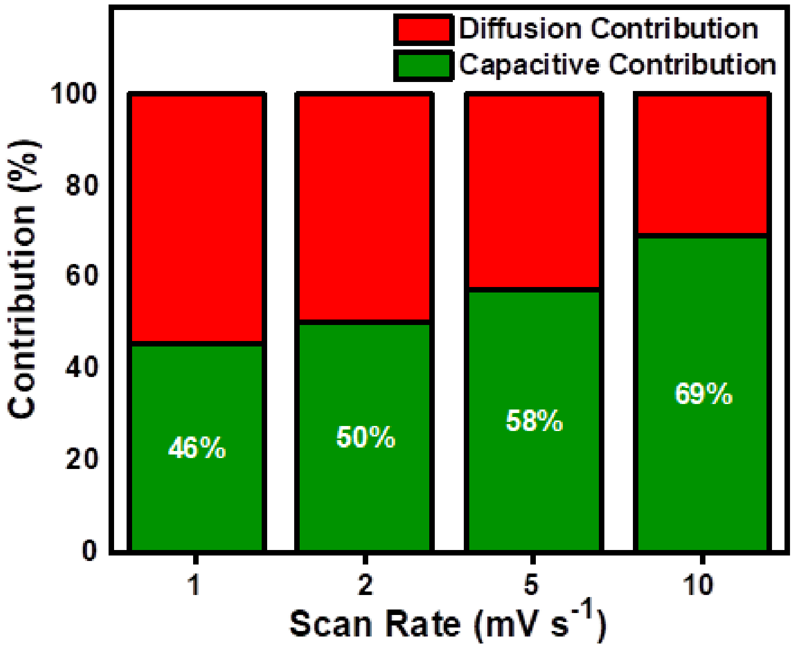

Supplementary Fig. 14 capacitive-controlled process contributions of d-V_2CT_x. The current density (i) in the CV curves can be separated into capacitive-controlled (k_1v) and diffusion-controlled ($k_2v^{1/2}$) contributions according to the equation[1]:

$$i(v) = k_1v + k_2v^{1/2}.$$

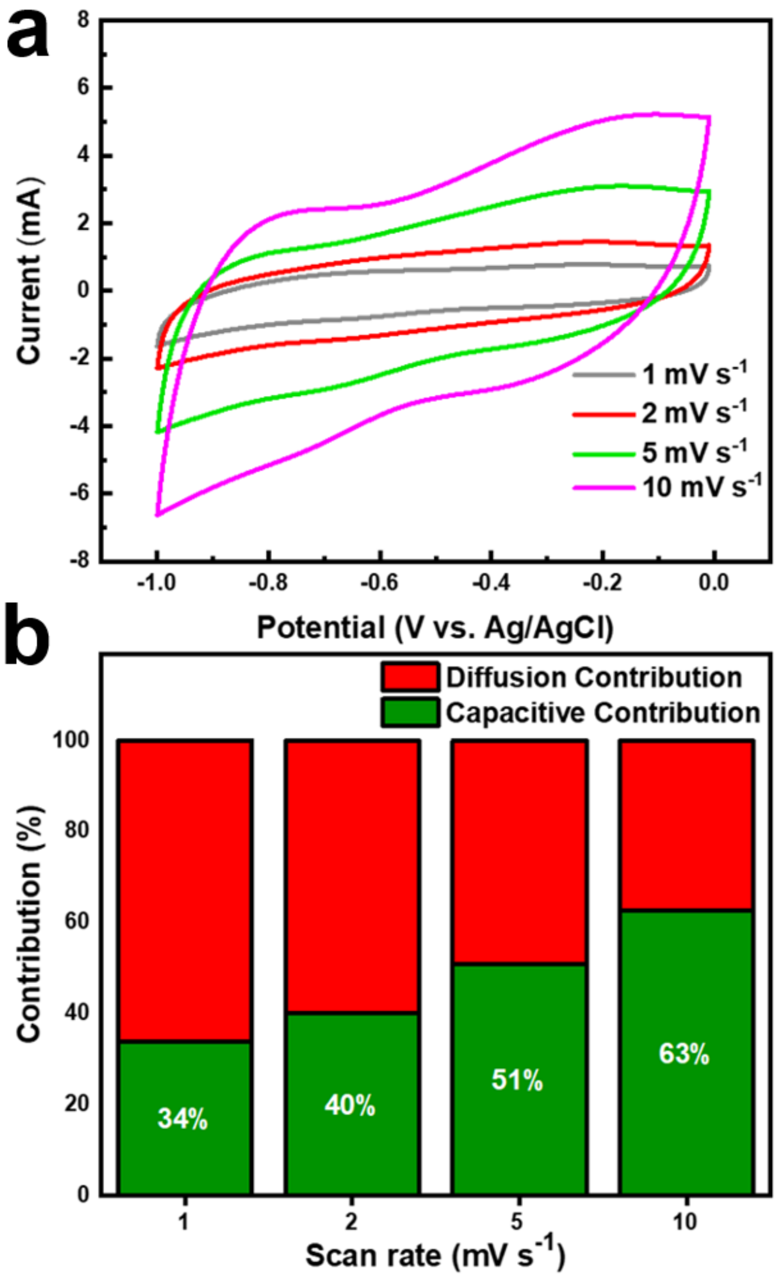

Supplementary Fig. 15 (a) CV curves at different scan rates and (b) the capacitive-controlled contribution of m-V_2CT_x.

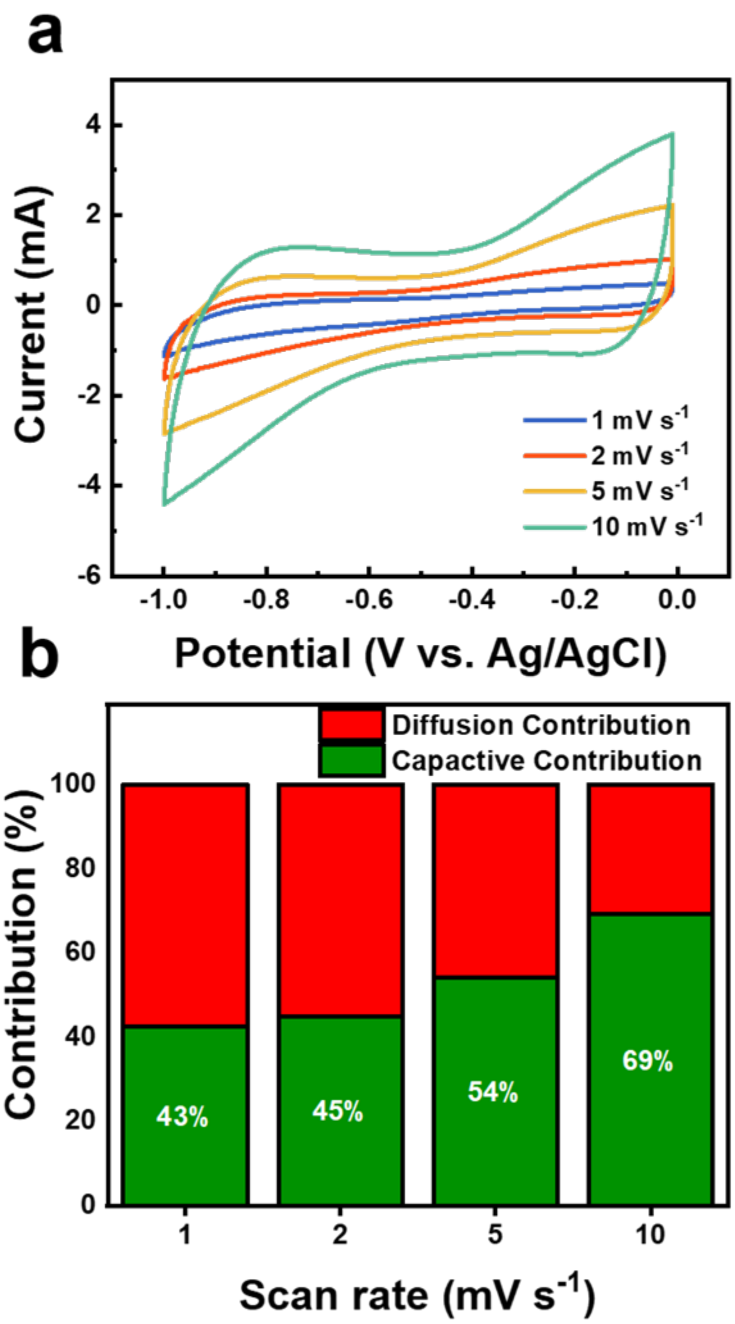

Supplementary Fig. 16 (a) CV curves at different scan rates and (b) the capacitive-controlled contribution of d-V_2CT_x in 0.25 M $(NH_4)_2SO_4$ electrolyte.

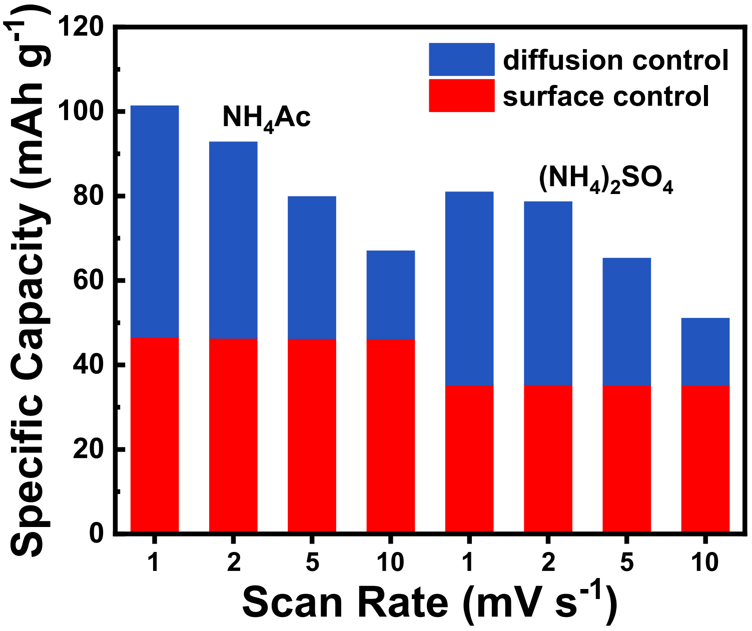

Supplementary Fig. 17 Contributions of d-V_2CT_x in 0.5 M NH_4Ac and 0.25 M $(NH_4)_2SO_4$ electrolytes, respectively.

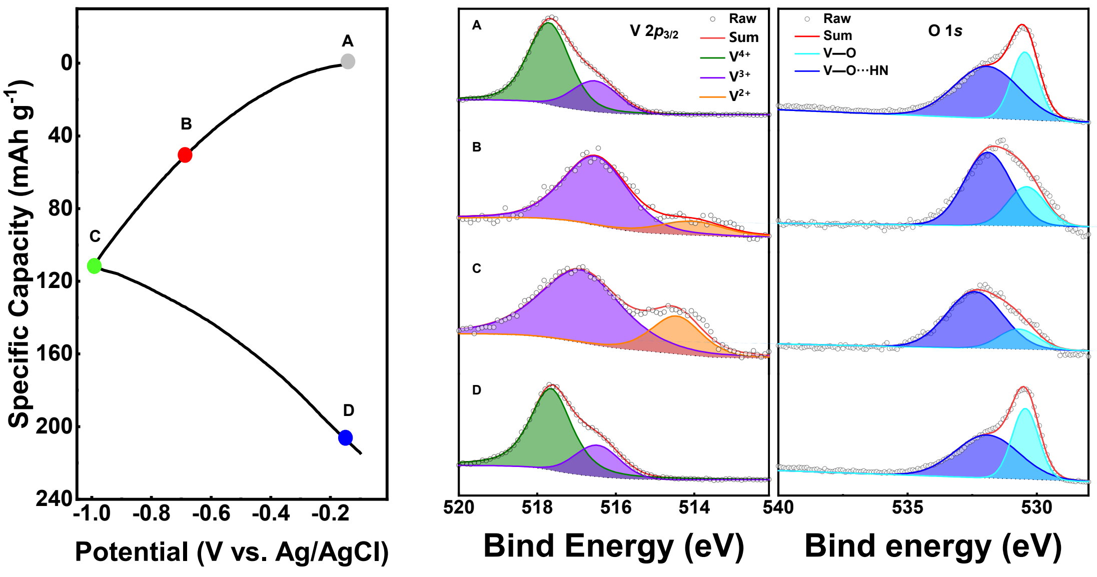

Supplementary Fig. 18 *Ex-situ* XPS spectra of d-V_2CT_x, V $2p_{3/2}$ and O 1*s*, respectively. The sample was obtained at the current density of 1 A g^{-1}.

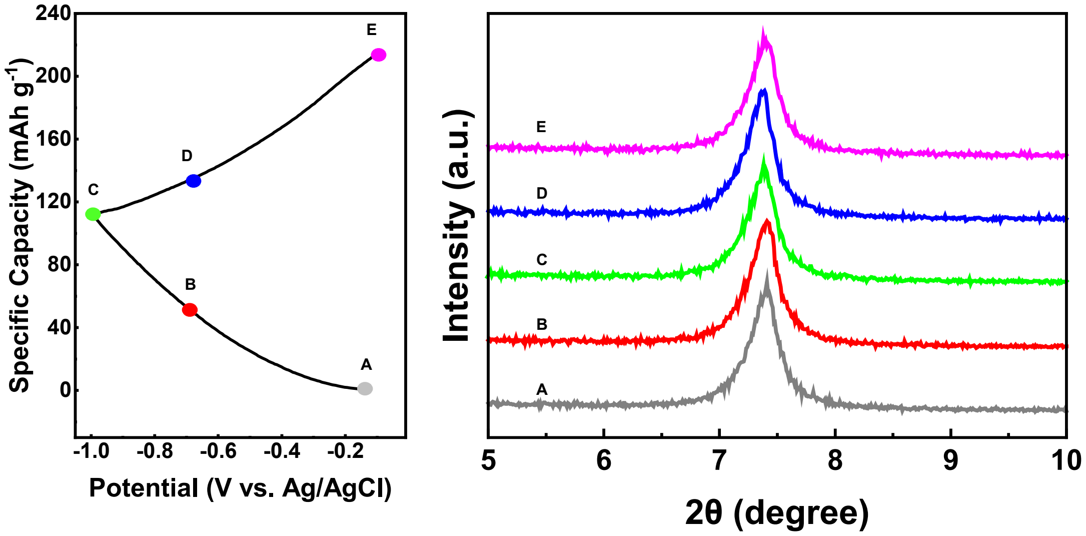

Supplementary Fig. 19 *Ex-situ* XRD patterns of d-V_2CT_x. The sample was obtained at the current density of 1 A g^{-1}.

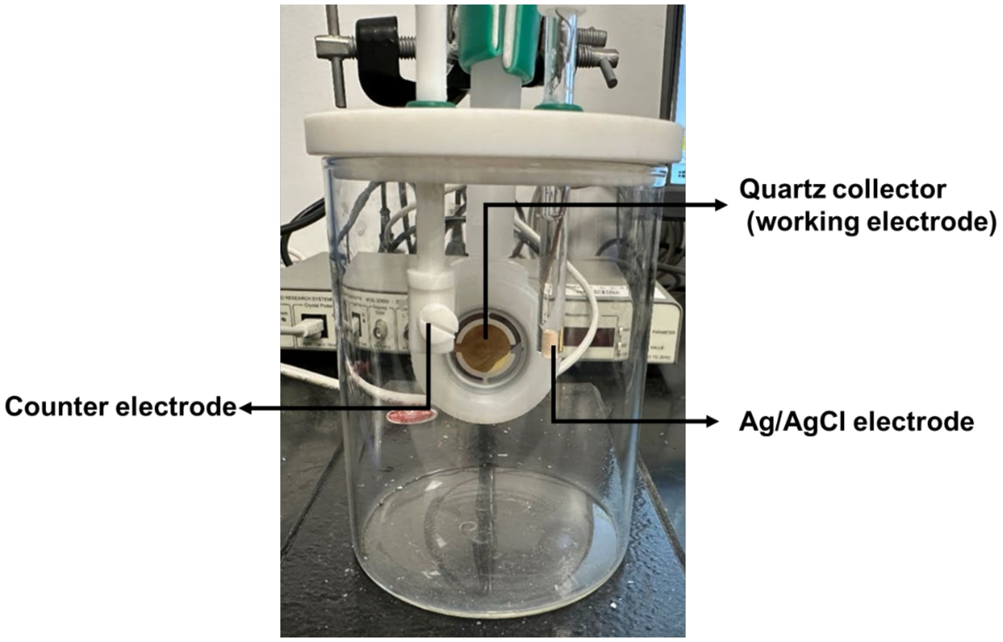

Supplementary Fig. 20 Three-electrode cell for *in-situ* EQCM measurement, in which MXene was coated on the quartz collector.

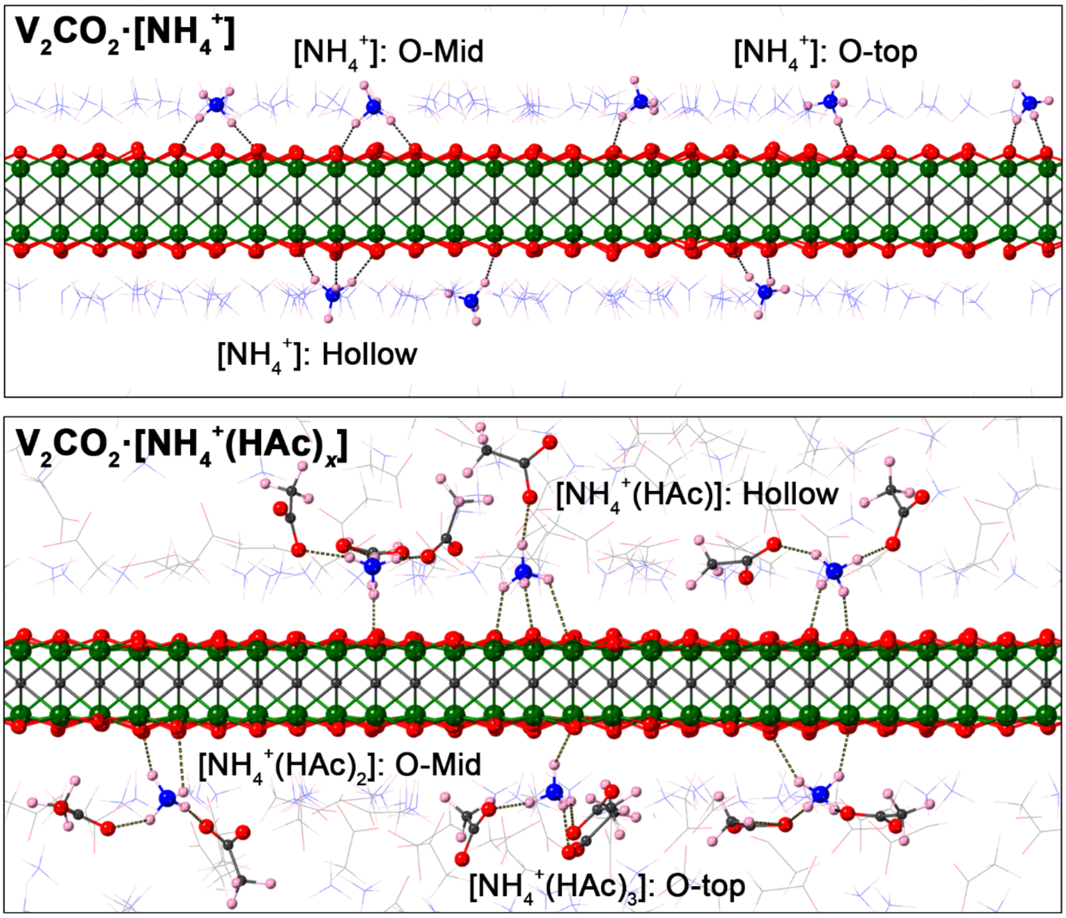

Supplementary Fig. 21 Typical adsorption configurations of $V_2CO_2 \cdot [NH_4^+]$ and $V_2CO_2 \cdot [NH_4^+(HAc)_x]$ models after a full relaxation of MD simulation by LAMMPS.

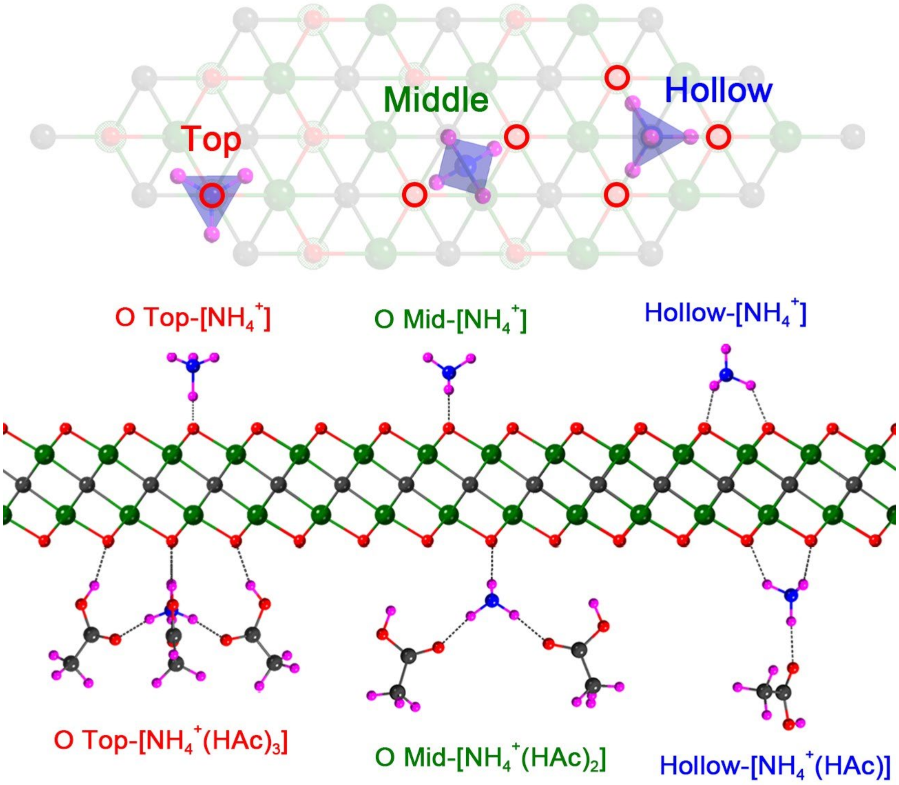

Supplementary Fig. 22 The top and front views of three models of V_2CT_x and $[NH_4^+(HAc)_x]$ groups.

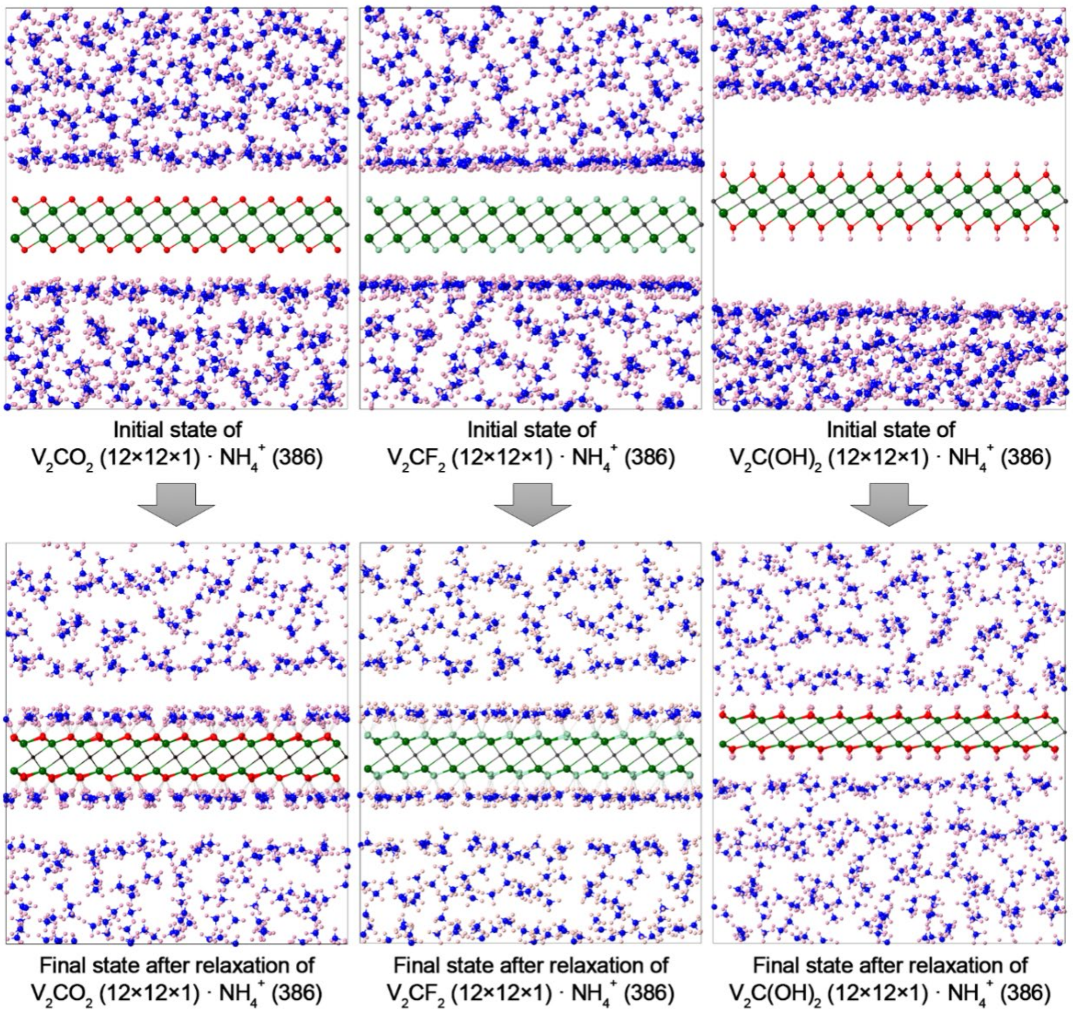

Supplementary Fig. 23 MD simulation of the adsorption behavior of NH_4^+ on the surface of V_2CT_x (T=-F, -O, -OH).

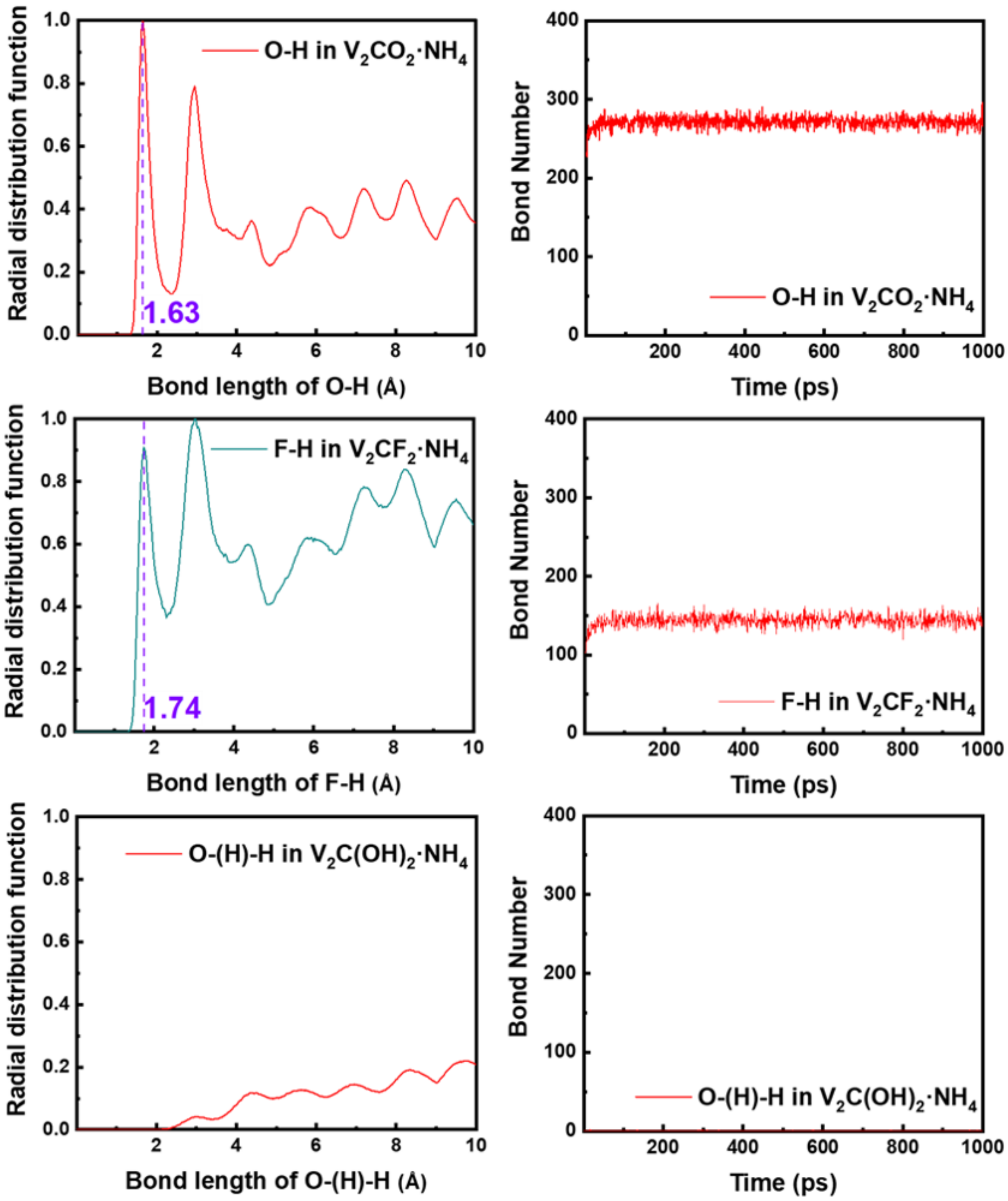

Supplementary Fig. 24 Bond length statistics from MD simulation with the adsorption of NH_4^+ on the surface of V_2CT_x (T=-F, -O, -OH).

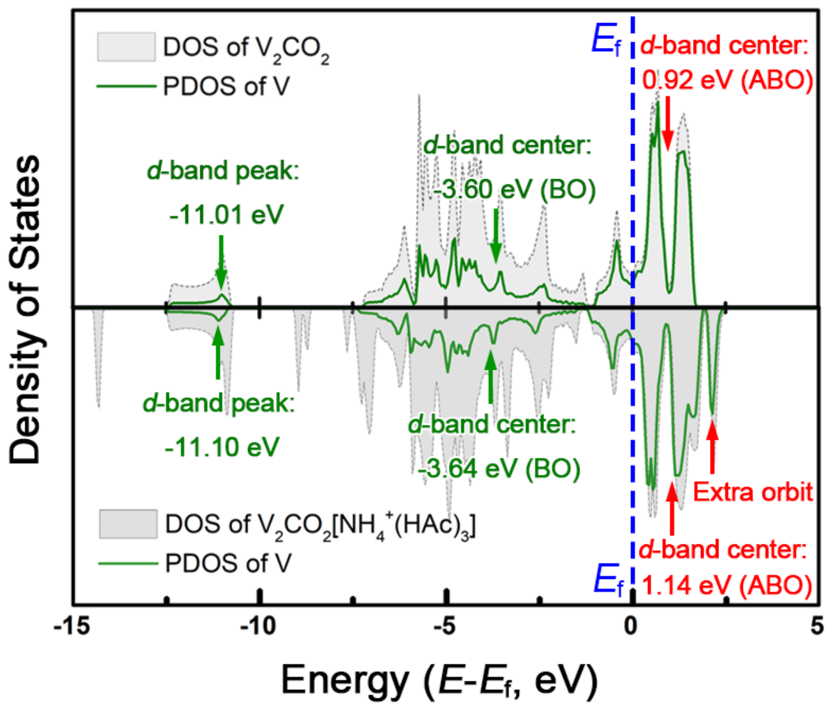

Supplementary Fig. 25 DOS analysis of V element in V_2CO_2 and $V_2CO_2[NH_4^+(HAc)_3]$.

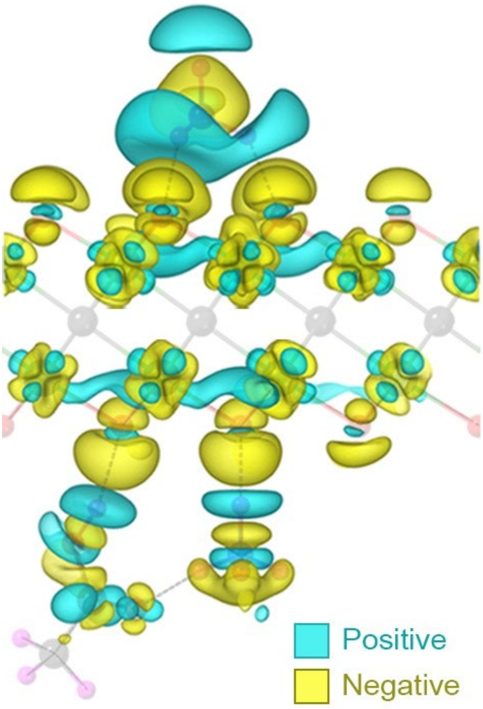

Supplementary Fig. 26 Charge density difference of $V_2CO_2[NH_4^+]$ and $V_2CO_2[NH_4^+(HAc)_3]$.

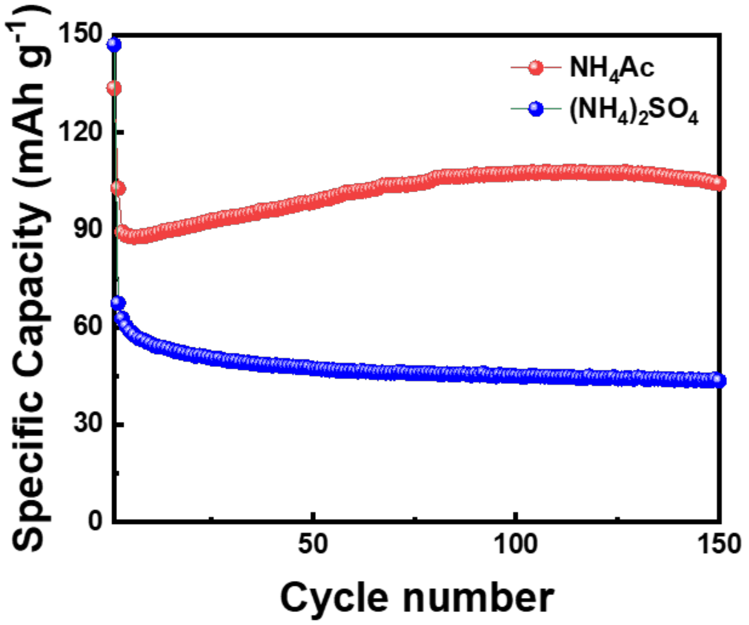

Supplementary Fig. 27 Cycling performances of 1T-MoS_2 in 0.5 M NH_4Ac and 0.25 M $(NH_4)_2SO_4$ electrolytes at 1 A g^{-1}. The test used Swagelok-type cells with the 1T-MoS_2 served as the working electrode, activated carbon as the counter electrode, and a saturated Ag/AgCl electrode acted as the reference electrode, respectively.

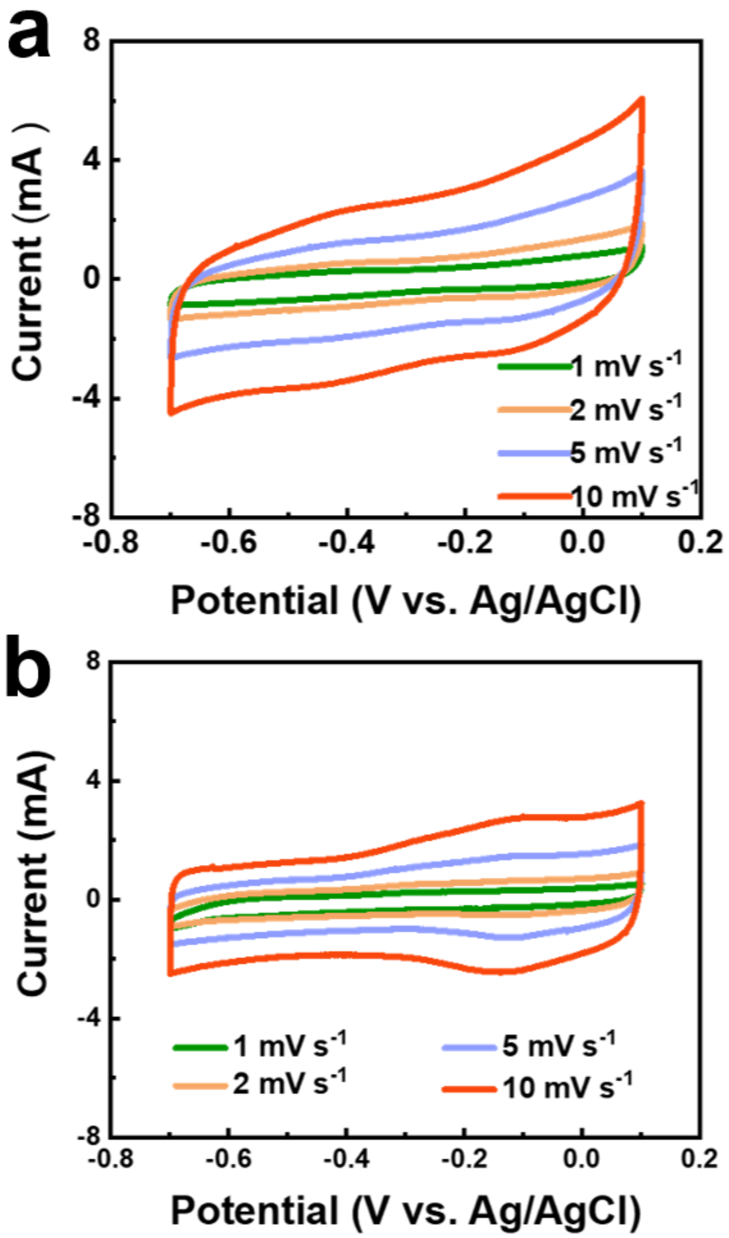

Supplementary Figure. 28 CV curves of 1T-MoS_2 in (a) 0.5 M NH_4Ac and (b) 0.25 M $(NH_4)_2SO_4$ electrolyte at different scan rates.

Supplementary Table 1 force field parameters considered in the MD simulations

Pair Coefficient	D_0 (kcal/mol)	R_0 (Å)
V, in MXene	0.0266000000	3.1440000000
C, in MXene	0.1050000000	3.8510000000
O, in MXene	0.2280000124	2.8597848722
C, in methyl group	0.0389999952	3.8754094636
C, in charged carboxylate group	0.1479999981	3.6170487995
H, bonded to carbon	0.0000000000	0.0000000000
H, bonded to nitrogen	0.0000000000	0.0000000000
O, in charged carboxylate group	0.2280000124	2.8597848722
N, with 4 substituents	0.1669999743	3.5012320066
F, in MXene	0.0687685101	3.0808078941
**Bond Coefficient**	k_1 **(kcal/mol Å^2)**	r_0 **(Å)**
V-C	560.0000	2.4100
V-O	560.0000	2.1200
C-C	283.0924	1.5200
C-H	340.6175	1.1050
C-O	540.0000	1.2500
H-N	457.4592	1.0260
V-F	560.0000	2.0600
**Angle Coefficient**	k_2 **(kcal/mol rad^2)**	θ_0 **(deg)**
C-V-C	30.0000	95.5200
C-V-O	30.0000	87.2900
O-V-O	70.0000	109.5000
V-C-V	30.0000	109.4700
V-O-V	60.0000	109.5000
C-C-H	45.0000	109.5000
H-C-H	39.5000	106.4000
C-C-O	68.0000	120.0000

O-C-O	145.0000	123.0000
H-N-H	36.0000	105.5000
C-V-F	30.0000	87.2900
V-F-V	60.0000	109.4700

Supplementary Reference

1 Pan QG, Zheng YP, Tong ZP, Shi L, Tang YB. Novel Lamellar Tetrapotassium Pyromellitic Organic for Robust High-Capacity Potassium Storage. *Angew. Chem. Int. Ed.* **60**, 11835-11840 (2021).
