# Supplementary material for: An acetate electrolyte for enhanced pseudocapacitve capacity in aqueous ammonium ion batteries
Source: Nat Commun. 2024 Mar 2;15:1934. doi: 10.1038/s41467-024-46317-5 (PMC10908845; doi:10.1038/s41467-024-46317-5)
Supplement: Supplementary file 3 — Inventory of Supplementary Information [file 41467_2024_46317_MOESM3_ESM.pdf]

Inventory of Supplementary Information

An acetate electrolyte for enhanced pseudocapacitve capacity in aqueous ammonium ion batteries

Zhuoheng Bao[1], Chengjie Lu[1], Qiang Liu[1], Fei Ye[1], Weihuan Li[1], Yang Zhou[1], Long Pan[1], Lunbo Duan[2], Hongjian Tang[2], Yuping Wu[2], Linfeng Hu[1]*, ZhengMing Sun[1]*

Supplementary Fig. 1-28

Supplementary Table 1

Supplementary Information Reference

---

[1] School of Materials Science and Engineering, Southeast University, Nanjing, 211189, China

These authors contributed equally: Zhuoheng Bao, Chengjie Lu

Address correspondence to: linfenghu@seu.edu.cn; zmsun@seu.edu.cn

[2] School of Energy and Environment, Southeast University, Nanjing 211189, P. R. China
