# Supplementary material for: An acetate electrolyte for enhanced pseudocapacitve capacity in aqueous ammonium ion batteries
Source: Nat Commun. 2024 Mar 2;15:1934. doi: 10.1038/s41467-024-46317-5 (PMC10908845; doi:10.1038/s41467-024-46317-5)
Supplement: Supplementary file 4 — Reporting Summary [file 41467_2024_46317_MOESM4_ESM.pdf]

nature portfolio

Corresponding author(s): Lifeng Hu, ZhengMing Sun

Last updated by author(s): Feb 11, 2024

Reporting Summary

Nature Portfolio wishes to improve the reproducibility of the work that we publish. This form provides structure for consistency and transparency in reporting. For further information on Nature Portfolio policies, see our Editorial Policies and the Editorial Policy Checklist.

Statistics

For all statistical analyses, confirm that the following items are present in the figure legend, table legend, main text, or Methods section.

n/a	Confirmed	
☐	☒	The exact sample size (*n*) for each experimental group/condition, given as a discrete number and unit of measurement
☐	☒	A statement on whether measurements were taken from distinct samples or whether the same sample was measured repeatedly
☒	☐	The statistical test(s) used AND whether they are one- or two-sided<br>*Only common tests should be described solely by name; describe more complex techniques in the Methods section.*
☒	☐	A description of all covariates tested
☒	☐	A description of any assumptions or corrections, such as tests of normality and adjustment for multiple comparisons
☒	☐	A full description of the statistical parameters including central tendency (e.g. means) or other basic estimates (e.g. regression coefficient) AND variation (e.g. standard deviation) or associated estimates of uncertainty (e.g. confidence intervals)
☒	☐	For null hypothesis testing, the test statistic (e.g. *F*, *t*, *r*) with confidence intervals, effect sizes, degrees of freedom and *P* value noted<br>*Give P values as exact values whenever suitable.*
☒	☐	For Bayesian analysis, information on the choice of priors and Markov chain Monte Carlo settings
☒	☐	For hierarchical and complex designs, identification of the appropriate level for tests and full reporting of outcomes
☒	☐	Estimates of effect sizes (e.g. Cohen's *d*, Pearson's *r*), indicating how they were calculated

Our web collection on statistics for biologists contains articles on many of the points above.

Software and code

Policy information about availability of computer code

Data collection	Commercial softwares utilized: MedeA 3.1 and VASP 5.4.4 (for DFT calculations); LAMMPS (for MD simulations).
Data analysis	CrystalMaker 10, Igor 8, and Vesta (open source) were utilized for creating models and analyzing data of calculations.

For manuscripts utilizing custom algorithms or software that are central to the research but not yet described in published literature, software must be made available to editors and reviewers. We strongly encourage code deposition in a community repository (e.g. GitHub). See the Nature Portfolio guidelines for submitting code & software for further information.

Data

Policy information about availability of data

All manuscripts must include a data availability statement. This statement should provide the following information, where applicable:

- Accession codes, unique identifiers, or web links for publicly available datasets
- A description of any restrictions on data availability
- For clinical datasets or third party data, please ensure that the statement adheres to our policy

All data supporting the findings of this study are available in the article and its Supplementary Information.

Research involving human participants, their data, or biological material

Policy information about studies with human participants or human data. See also policy information about sex, gender (identity/presentation), and sexual orientation and race, ethnicity and racism.

Reporting on sex and gender	This research does not involve sex or gender based analyses.
Reporting on race, ethnicity, or other socially relevant groupings	This research does not involve reporting on race, ethnicity, or other socially relevant groupings.
Population characteristics	This research does not involve population characteristics.
Recruitment	This research does not involve recruitment.
Ethics oversight	This research does not involve ethics oversight.

Note that full information on the approval of the study protocol must also be provided in the manuscript.

Field-specific reporting

Please select the one below that is the best fit for your research. If you are not sure, read the appropriate sections before making your selection.

☐ Life sciences ☐ Behavioural & social sciences ☒ Ecological, evolutionary & environmental sciences

For a reference copy of the document with all sections, see nature.com/documents/nr-reporting-summary-flat.pdf

Ecological, evolutionary & environmental sciences study design

All studies must disclose on these points even when the disclosure is negative.

Study description	Ammonium ion batteries are appealing candidates for next-generation sustainable energy storage with the merits of low cost, inherent security, environmental friendliness, and excellent electrochemical properties. Herein, we utilized density functional theory (DFT) calculations and molecular dynamics (MD) simulations to explore the potential of V2CTx MXene as a promising anode candidate with a low working potential window. We further demonstrated that V2CTx MXene is a pseudocapacitive typed anode material for ammonium ion storage with a high specific capacity and an excellent capacity retention. In particular, we discovered that the profound pseudocapacitive storage behavior could only be observed in the ammonium acetate (NH4Ac) electrolyte. Benefiting from this unique acetate ion enhancement effect, the specific capacity of our V2CTx MXene surpasses all of the capacitive-typed electrodes in ammonium-ion batteries up to date. The findings in this work open a new door to realizing high capacity on sustainable ammonium ion storage by the acetate ion enhancement effect. It also makes a breakthrough in the capacity limitation of capacitive energy storage for both Faradaic and non-Faradaic types.
Research sample	The samples include V2CTx and 1T-MoS2, two kinds of inorganic two-dimensional materials. The V2CTx MXene was prepared through the sintering and etching of inorganic materials.1T-MoS2 was prepared by hydrothermal method. Details are described in the method section.
Sampling strategy	First, the samples were mixed with a conductive agent and binder in in N-methylpyrrolidone to form a slurry. Then, the slurry was sprayed on carbon paper and vacuum-dried. The obtained carbon paper was punched into small disks with a diameter of 1.0 cm with a sample mass loading of ~1.3 mg cm−2. The electrochemical measurements of the V2CTx MXene were carried out in the three-electrode Swagelok cell. Details are described in the method section.
Data collection	The crystal structure characteristics were studied by X-ray diffraction (XRD, Bruker D8 X-ray diffractor with Cu Kα radiation (λ = 1.5406 Å)). The morphology and structure of samples were characterized using scanning electron microscopy (SEM, Sirion 200) and transmission electron microscopy (TEM, FEI Talos F200X). The elemental composition and chemical state of samples were measured by X-ray photoelectron spectrum (XPS, Thermo Scientific Escalab 250Xi). The nitrogen adsorption and desorption isotherms were recorded by a Quanta Autosorb-IQ2 analyser. The cyclic voltammetry (CV) and electrochemical impedance spectroscopy (EIS) tests were conducted on an electrochemical workstation (CHI660E). The galvanostatic charge-discharge (GCD) and long-term cycling tests were recorded on a LAND battery test system (CT3001A). First-principles calculation together with molecular dynamics simulation was performed in this work. Those softwares were utilized: MedeA 3.1 and VASP 5.4.4 (for DFT calculations); LAMMPS (for MD simulations).<br>Z. B., Q. L. performed materials synthesis, battery assembly, and performance measurement. Z. B. and Q. L. performed SEM, TEM, XRD, and XPS. C. L. performed DFT calculations. W. J. and Y. Z. performed MD simulations.<br>Details are described in the method section.
Timing and spatial scale	The preparation time for the materials is around 1 to 3 days, while the electrode preparation time is around 1 to 2 days. The testing of electrochemical performance varies depending on the testing parameters, with testing periods ranging from 1 day to 1 month.
Data exclusions	There was no data excluded from the analyses.

Reproducibility	All conclusions are reproducible in the laboratory. The excellent electrochemical performance can be achieved in materials prepared from different batches. The chemicals involved in this experiment are commonly used, ensuring excellent reproducibility.
Randomization	Randomization was not necessary for this research.
Blinding	Blinding was not necessary for this research.

Did the study involve field work? ☐ Yes ☒ No

Reporting for specific materials, systems and methods

We require information from authors about some types of materials, experimental systems and methods used in many studies. Here, indicate whether each material, system or method listed is relevant to your study. If you are not sure if a list item applies to your research, read the appropriate section before selecting a response.

Materials & experimental systems

n/a	Involved in the study
☒	☐ Antibodies
☒	☐ Eukaryotic cell lines
☒	☐ Palaeontology and archaeology
☒	☐ Animals and other organisms
☒	☐ Clinical data
☒	☐ Dual use research of concern
☒	☐ Plants

Methods

n/a	Involved in the study
☒	☐ ChIP-seq
☒	☐ Flow cytometry
☒	☐ MRI-based neuroimaging

Plants

Seed stocks	This research does not involve seed stocks.
Novel plant genotypes	This research does not involve novel plant genotypes.
Authentication	Authentication was not necessary for this research.
